# Assessing Clinical Effects of Traditional Chinese Medicine Interventions: Moving Beyond Randomized Controlled Trials

**DOI:** 10.3389/fphar.2021.713071

**Published:** 2021-09-07

**Authors:** Xin Sun, Ling Li, Yanmei Liu, Wen Wang, Minghong Yao, Jing Tan, Yan Ren, Ke Deng, Yu Ma, Yuning Wang, Jin Chen, Wei Huang, Qing Xia, Youping Li, Hongcai Shang

**Affiliations:** ^1^ Chinese Evidence-Based Medicine Center, West China Hospital, Sichuan University, Chengdu, China; ^2^ Department of Integrated Traditional Chinese and Western Medicine, West China Hospital, Sichuan University, Chengdu, China; ^3^ Key Laboratory of Chinese Internal Medicine of Ministry of Education, Dongzhimen Hospital, Beijing University of Chinese Medicine, Beijing, China

**Keywords:** traditional Chinese medicine, clinical effects, clinical research methodology, integrative medicine, Chinese patent medicine

## Introduction

Traditional Chinese medicine (TCM) is a feature of Chinese civilization and has made significant contributions to development of the Chinese. Nowadays, TCM has attracted extensive attentions across the country and the support for TCM has become part of the national strategy (Anonymous, 2019; Anonymous, 2021). A fundamental policy of China is that equal importance is attached to TCM and western medicine. In the treatment of diseases, for instance the fight against COVID-19, TCM and western medicine are often used in an integrated medicine approach (Chan et al., 2020; Du et al., 2020; Ren et al., 2020), in which the strengths of TCM with modern medicine (western medicine) are combined to improve clinical effects of healthcare interventions (Wang and Xu, 1997; Anonymous, 2015). In particular, the unique features of TCM–holism and treatment based on syndrome identification–are demonstrated in combination with practice of western medicine.

Over the past decades, this practice pattern received wide acceptance in the Chinese healthcare system and the unique roles of TCM in the diseases prevention and treatment, for example acupuncture for migraine prophylaxis (Zhao et al., 2017) and stress urinary incontinence treatment (Liu et al., 2017), are increasingly recognized by the international community (Anonymous, 2020). However, TCM interventions are a highly complex system. The prescription of TCM interventions is usually individualized that is based on four theoretical foundations, including the combination of syndrome and disease for diagnosis, dynamic evolution of disease syndromes, treatment based on syndrome identification, and the integrated *principle-method-recipe-medicines* approach. Under these theoretical framework, dynamic changes of syndromes would require changes in prescription (Shi et al., 2021).

The emergence of evidence-based medicine brings new ideas and methods–such as randomized controlled trials (RCTs) and systematic reviews–to the generation of evidence on clinical effects (Djulbegovic and Guyatt, 2017; Tian et al., 2021). However, clear differences in theoretical bases and underlying philosophies between TCM and western medicine inevitably presents significant challenges in assessing clinical effects of TCM interventions. The development of clinical research methodologies that specifically address clinical effects of TCM is vital to the innovation and thriving of TCM and represents a key strategy for TCM modernization (Wang and Huang, 2019). In this article, we discussed potential strategies, proposed an integrated approach that may effectively address challenges, and reported our practices in the application of the integrated approach for establishing clinical effects of post-approval Chinese patent medicines.

## Assessing Clinical Effects of TCM Interventions in the Context of Integrative Medicine: Moving Beyond Randomized Controlled Trials

Evidence-based medicine framework usually treats classical RCTs (i.e., explanatory RCTs) as the gold standard, and combines other research methods (e.g., cohort studies) to develop a hierarchy of evidence (Centre for Evidence-Based Medicine, 2011). However, due to complexity of TCM interventions and its differences from western medicine, it is unlikely that RCTs can fully reflect features of TCM interventions. In the past decades, efforts from the methodology community were mainly centered on therapeutic efficacy; this focus may result that other equally important questions such as effectiveness or safety in real-world clinical setting are less attended. Additionally, this research pattern–intensive focus on classical RCT–often has limitations in integrating complex theories and practical features of TCM interventions. The resulting findings may not reflect the nature of TCM interventions. Therefore, an enhanced approach is strongly desirable.

In addressing complexities of TCM interventions, one strategy is standardization of important diagnosis and treatment factors that have potential impacts on the estimates of treatment effects of TCM interventions. In view of the complexities of TCM interventions and challenges of standardizing TCM features (e.g., syndromes), a progressive standardization strategy may be applied when assessing clinical effects of TCM interventions, that is, first achieve standardization of those fully or largely standardizable factors, then partially standardizable factors. For example, the assessment of clinical effects of TCM interventions may be based on three different disease diagnoses models, including disease diagnosis using western medicine theory, diagnosis using TCM-based syndrome differentiation, or a combination of both (Anonymous, 2015). While the first is often standardized, the complexity of TCM interventions may not be fully represented. Diagnosis of diseases purely based on TCM theory is often variable and less standardized. In such case, a diagnosis may be made primarily based on western medicine, and the addition of disease differentiation using TCM theory may strengthen the feature of TCM interventions.

The other strategy is the development of innovative methods, in addition to explanatory RCTs, to embrace the complexities of TCM interventions, since standardization is unlikely applicable to all cases or scenarios of TCM interventions. In addressing the diversity of TCM interventions, it is necessary to build a problem-oriented methodology approach that encompasses all relevant research questions about TCM interventions and addresses research needs at different stages or phases.

## Developing an Integrated Methodological Approach for Assessing TCM Interventions: Focusing on Standardizable TCM Interventions

Acupuncture and Chinese patent medicines are commonly used therapeutic modalities in integrative medicine. They are highly standardizable and widely recognized interventions both in China and abroad. However, even for such therapeutic modalities, the applicable methodologies for assessing their treatment effects still differ with those for western medicine. The key question about acupuncture and Chinese patent medicine is how their evidence contains features of TCM while treatment effects are reliably assessed.

Although classical RCTs carry strong interval validity, they are never enough largely due to its restrictions in study factors (e.g., setting, population and interventions) and the resulting limitations to reflect TCM features. The last two decades have witnessed the rise of practice-based research, whereby real-world data generated from routine practice are primarily used for research (Sherman et al., 2016; Sun et al., 2018). This feature particularly fits for the characteristics of acupuncture and Chinese patent medicines, in which practical variations are allowed while standardization is feasible. However, such studies are often observational in nature and are subject to bias.

**FIGURE 1:**
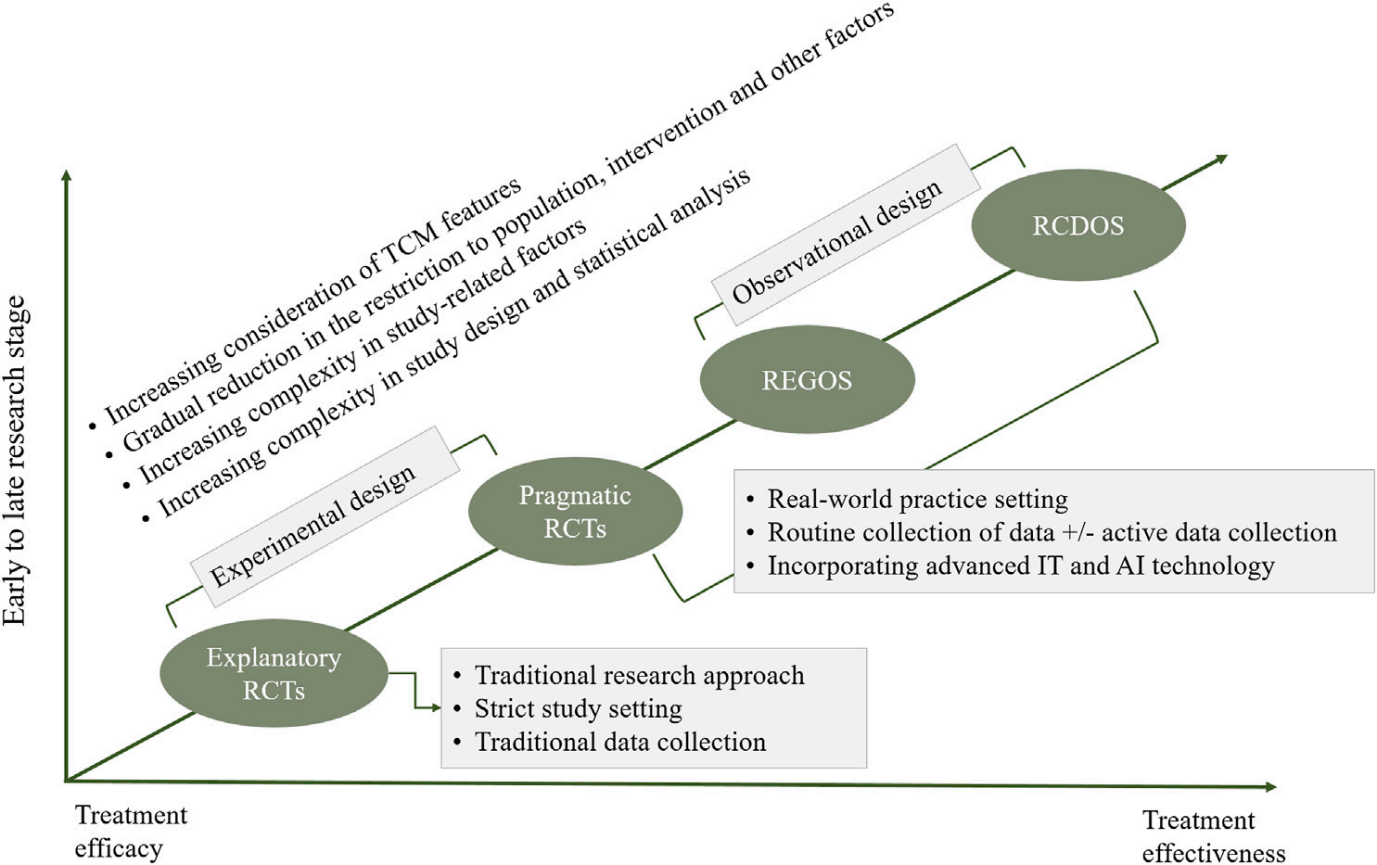
Integrated approach for assessing clinical effects of traditional Chinese medicine (i.e., 4R approach). Notes: eRCT, explanatory randomized controlled trial; pRCT, pragmatic randomized controlled trial; RCDOS, observational studies using routinely collected data; REGOS, observational studies using patient registry data; IT, information technology; AI, artificial intelligence.

In addressing the limitations of the current approaches, we propose an integrated methodological approach for assessing clinical effects of acupuncture and Chinese patent medicines (eRCT-pRCT-RCDOS-REGOS, 4R, Figure 1). Explanatory trials and studies at real-world practice setting are merged through a stepwise manner, and research questions at different stages–e.g., efficacy versus effectiveness–are addressed. In the two extremes between explanatory trials and observational studies with routinely collected data, the TCM features are increasingly involved. Consequently, all these four research methods, in a collective manner, contribute to reliable and comprehensive assessment of clinical effects of acupuncture and Chinese patent medicines.

Within this integrated methodological approach, explanatory randomized controlled trials (eRCTs) may be used at early stage to establish efficacy of acupuncture and Chinese patent medicine. Although rigorous in design and having relatively low risk of bias, it may contain fewer features of TCM. Once the efficacy is established, research at real-world practice setting–that allows more flexible inclusion of study population and interventions–would be conducted. Pragmatic randomized controlled trials (pRCTs) represent an important vehicle to establish effectiveness of acupuncture and Chinese patent medicines in the real-world settings. This method integrates more features of TCM interventions, and combines advantages of explanatory trials (e.g., randomization), and such, it may be an ideal option for assessing clinical effectiveness of TCM interventions (Zuidgeest et al., 2017; Shi et al., 2021).

Observational studies using routinely collected data (RCDOS) or observational studies using patient registry data (REGOS) are two research approaches alternative to pRCTs that may be used for assessing clinical effects of acupuncture and Chinese patent medicines. Despite their significant strengths in collection of real-world practice data, these two approaches often suffer from serious limitations due to the nature of observational designs. Nevertheless, they may offer irreplicable advantages in assessing syndrome-driven treatment heterogeneity, understanding changes of treatment pattern, assessing real-world safety particularly those rare adverse reactions, exploring component effect in case of complex interventions, investigating interactions between western medicine and TCM, and predicting clinical effects of individual patients.

Across all these methods, one critical issue is how to infuse the theory and features of TCM into methodological components. Despite relatively high degree of standardization when using acupuncture and Chinese patent medicine, objective complex factors continue to present challenges in assessing clinical effects, including outcome measurements that are applicable to TCM features (Wang and Huang, 2019; Zhang et al., 2021), effect estimation in rare event setting (Greenland et al., 2016), study designs involving patient preferences (Angell, 1984; Rücker, 1989; Cameron et al., 2018), repeated measures in TCM setting (Albert, 1999; Twisk, 2004; Goetgeluk and Vansteelandt, 2008; Wang et al., 2012), causal inference of treatment effects in complex design and data settings (Zigler and Dominici, 2014; Koch et al., 2020), and decomposition of effects in complex intervention setting. Continuing efforts are warranted to address these issues.

## Use of Integrated Approach for Assessing Clinical Effects of Post-Approval Chinese Patent Medicines: Practical Examples

**FIGURE 2:**
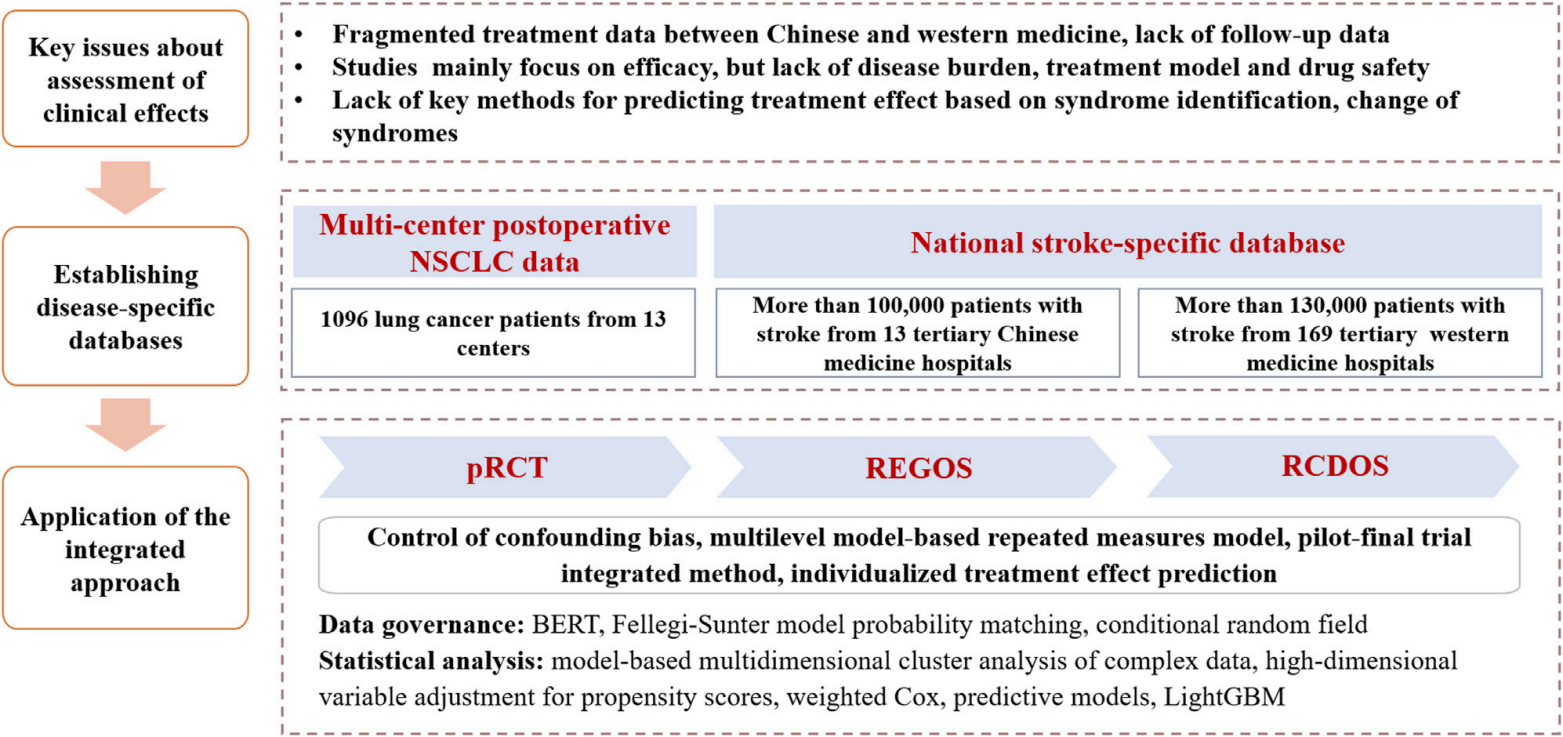
Use of the integrated approach for assessing clinical effects of post-approval Chinese patent medicines. Notes: NSCLC, non-small cell lung cancer; pRCT, pragmatic randomized controlled trial; REGOS, observational studies using patient registry data; RCDOS, observational studies using routinely collected data.

Post-approval Chinese patent medicines are often faced with important evidence gaps. Because the approval of Chinese patent medicines usually relies on traditional clinical trials, the resulting findings are often subject to restrictions in study designs. Therefore, post-approval studies of Chinese patent medicines are critical. Such studies usually aim to establish clinical effects in the context of TCM theory, whereby TCM features are increasingly present. These may include effectiveness, long-term effects, safety and heterogeneity in effects among subpopulations with varying characteristics (e.g., differential syndromes). In the past years, efforts have been made to apply the proposed approach for assessing post-approval Chinese patent medicines among selected diseases, for which TCM interventions may have special advantages (Figure 2).

For example, stroke is characterized by high morbidity, disability, mortality, recurrence and economic burden (Virani et al., 2020). Although treatments with western medicine, such as intravenous thrombolysis and mechanical thrombectomy, have clear effect on patients with acute ischemic stroke, limited treatment options are available for patients with ischemic stroke in recovery and sequelae stages (Powers et al., 2018). Early evidence suggested efficacy of Chinese patent medicines on the lowering of stroke complications and improvement of stroke sequelae (Li et al., 2017; Liang et al., 2018; Liu et al., 2018). However, there continues to be major evidence gaps, including effectiveness and safety and differential effects by varying TCM features among patients in real-world clinical setting.

In our efforts to address these evidence needs, we have developed two stroke-specific databases, including one multicenter stroke-specific registry database from TCM hospitals and one nationwide stroke-specific routinely collected healthcare database from western medicine hospitals. We initially applied data processing technologies and common data model to standardize the data. We then applied the integrated approach to undertake studies addressing a series of research questions about clinical effects of Chinese patent medicines, including investigation of disease burden and treatment patterns of stroke using cross-sectional design; understanding types of Chinese medicine syndromes and regional distribution of acute cerebral hemorrhage and acute cerebral infarction, using model-based multidimensional clustering analysis; assessment of effectiveness and safety of Chinese patent medicines (e.g., TCM injection on top of routine thrombolysis treatment) among patients at stroke sequelae, using retrospective cohort study design and casual model; and development of a model for predicting individual treatment effects among patients with Chinese patent medicines treatments, based on machine learning methods.

In another example, effective treatments of non-small cell lung cancer (NSCLC) usually include surgery, chemotherapy, radiotherapy, molecular targeting and immunotherapy (Postmus et al., 2017). However, drug resistance and adverse reactions are two major limitations of these treatment options, which, in return, seriously compromise quality of life of patients (Chinese Medical Association, 2020). TCM may have advantages in alleviating adverse reactions caused by typical western medicine and raising patient immunity, thus improving quality of life, and potentially prolonging survival (Li et al., 2019; Yang et al., 2020). Nevertheless, evidence is lacking.

In the efforts to address this important evidence gap, we established a multicenter postoperative NSCLC patient registry, whereby we applied the integrated approach to address issues about clinical effects of Chinese patent medicines for cancer patients. These included a multi-center pRCT that compared Zhongliuping 3 formula versus usual care to examine its effects on clinical symptoms and quality of life in NSCLC patients after surgery, in which multiple data sources were collected; a multi-center pRCT that compared Shenlingcao oral liquid versus usual care among NSCLC patients after receiving surgery to confirm its effect on quality of life and alleviation of serious adverse reactions caused by chemotherapy; and a cohort study using data from multicenter pRCTs to analyze long-term effectiveness, safety and heterogeneity in effects among different syndromes.

## Conclusion

In this paper, we proposed an integrated approach (i.e., 4R approach) that combines the strengths of classical RCTs and practice-based research, and infuses TCM theories and features into key study characteristics. Through this approach, the challenges may be effectively addressed in assessing clinical effects of TCM interventions. We also provided practical examples about the use of integrated approaches for assessing clinical effects of post-approval Chinese patent medicines in stroke and lung cancer patients. However, this approach has not been widely tested, and more applications are warranted. We are hopeful that the integrated approach may be further enhanced through wider efforts and become an accepted model in assessing clinical effects of TCM interventions.
